# Strong mucosal adhesion enhances the anti-infective effectiveness of T4-like phages

**DOI:** 10.1128/aem.02462-25

**Published:** 2026-06-04

**Authors:** Shujie Luo, Bei Zhou, Hauke Smidt, Yan Lin, Weiyun Zhu

**Affiliations:** 1 Laboratory of Gastrointestinal Microbiology, College of Animal Science and Technology, Nanjing Agricultural University, Nanjing, China; 2 National Center for International Research on Animal Gut Nutrition, Jiangsu Key Laboratory of Gastrointestinal Nutrition and Animal Health, Nanjing Agricultural University, Nanjing, China; 3 Laboratory of Microbiology, Wageningen University & Research 4508, Wageningen, the Netherlands; University of Nebraska-Lincoln, Lincoln, Nebraska, USA

**Keywords:** phage adherence, phage therapy, protective efficacy, immune response

## Abstract

Bacteriophage adhesion to intestinal mucus has been proposed as an important mechanism for preventing bacterial infection. However, whether differences in adhesion capacity among closely related phages translate into distinct protective outcomes remains unclear. In this study, we compared two T4-like bacteriophages, S143_2 and W143, to investigate how variation in mucosal adhesion influences antibacterial efficacy and host responses. Both phages exhibited comparable lytic activity against enteropathogenic *Escherichia coli* (EPEC143) but differed in adhesion capacity to intestinal epithelium. *In vitro* assays demonstrated that phages adhered to mucus-secreting IPEC-1 cells via a reversible mucus association, with S143_2 displaying significantly stronger adhesion than W143. This difference was further confirmed *in vivo*, where S143_2 showed higher mucosal retention and a significantly greater mucosa-to-digesta ratio of phage titers in the small intestine. Functionally, enhanced adhesion was associated with improved protective efficacy. In a prophylactic mouse model, pretreatment with S143_2 resulted in reduced body weight loss and lower intestinal pathogen load compared to W143. Furthermore, S143_2 induced a more robust systemic immune response evidenced by elevated serum cytokines and the enrichment of immune-related signaling pathways in the intestinal transcriptome. Together, these findings demonstrate that mucosal adhesion capacity is a critical determinant of phage performance *in vivo*. Our results highlight that, beyond host range and bactericidal capacity, adhesion properties should be considered a key criterion in the rational selection of bacteriophages for preventing and treating mucosa-associated infections.

**IMPORTANCE:**

Phages are among the most promising antibiotic alternatives, yet their evaluation has largely focused on bacterial host range and lytic activity. Our findings highlight mucosal adhesion as a previously overlooked but decisive factor in phage efficacy when targeting mucosa-associated infections. Phages with stronger epithelial adhesion exhibit superior protection and immune modulation in the gut, underscoring that adhesion capacity should be integrated as a key criterion in the rational selection and engineering of therapeutic phages.

## INTRODUCTION

The intestinal mucosal barrier serves as a critical defense mechanism against pathogenic bacterial infections, operating through a tripartite system encompassing chemical, physical, and microbial barrier functions (1). Recent studies have identified phages as a novel element of the microbial barrier, capable of effectively counteracting pathogen invasion (2, 3). Investigations on T4 phages have shown that they adhere to the intestinal mucosa through interactions between their Hoc proteins and MUC2 in mucus (4, 5). Once attached, phages display subdiffusive motion, thereby enhancing their encounter frequency with bacteria (6). Although Hoc–MUC2 interactions are likely a common feature, the critical binding sites vary among specific phages (7, 8). To date, however, no studies have examined whether T4 phages with distinct Hoc proteins differ in adhesion efficiency, or whether such variation translates into differential protective efficacy.

Furthermore, once adhered, phages may undergo epithelial uptake followed by intracellular degradation or transcytosis into the bloodstream, both of which can stimulate host immune responses (9–12). Prior studies have shown that immune responses to phages differ substantially, depending on the specific phage strains and the administration route (13–15). Yet, it is unclear whether structurally similar phage strains, recognized identically by the immune system, induce comparable immune responses.

To address these gaps, we selected two T4-like phages to investigate potential differences in their adhesion capacity, protective efficacy against bacterial invasion, and the immune responses induced following epithelial uptake or transcytosis. Compared with phage T4, S143_2 exhibits a query coverage of 48% and a genomic identity of 75.5%, whereas W143 shows a query coverage of 91% and a genomic identity of 95.4%. Phage S143_2 was obtained from the experimental animal facility of Nanjing Agricultural University (from a 63-day-old pig), whereas W143 was isolated from the intestinal contents of a pig in Zhenjiang, Jiangsu Province (from an 80-day-old pig). Both phages were derived from intestinal digesta and exhibited lytic activity against EPEC143. This study demonstrates that phage adhesion capacity variations can significantly influence *in vivo* antibacterial outcomes, thereby establishing a rational foundation for optimizing phage selection strategies in both preventive and therapeutic applications against bacterial infections.

## RESULTS

### Adhesion capacity and protective effect of different T4-like phages *in vitro*

**Fig 1:**
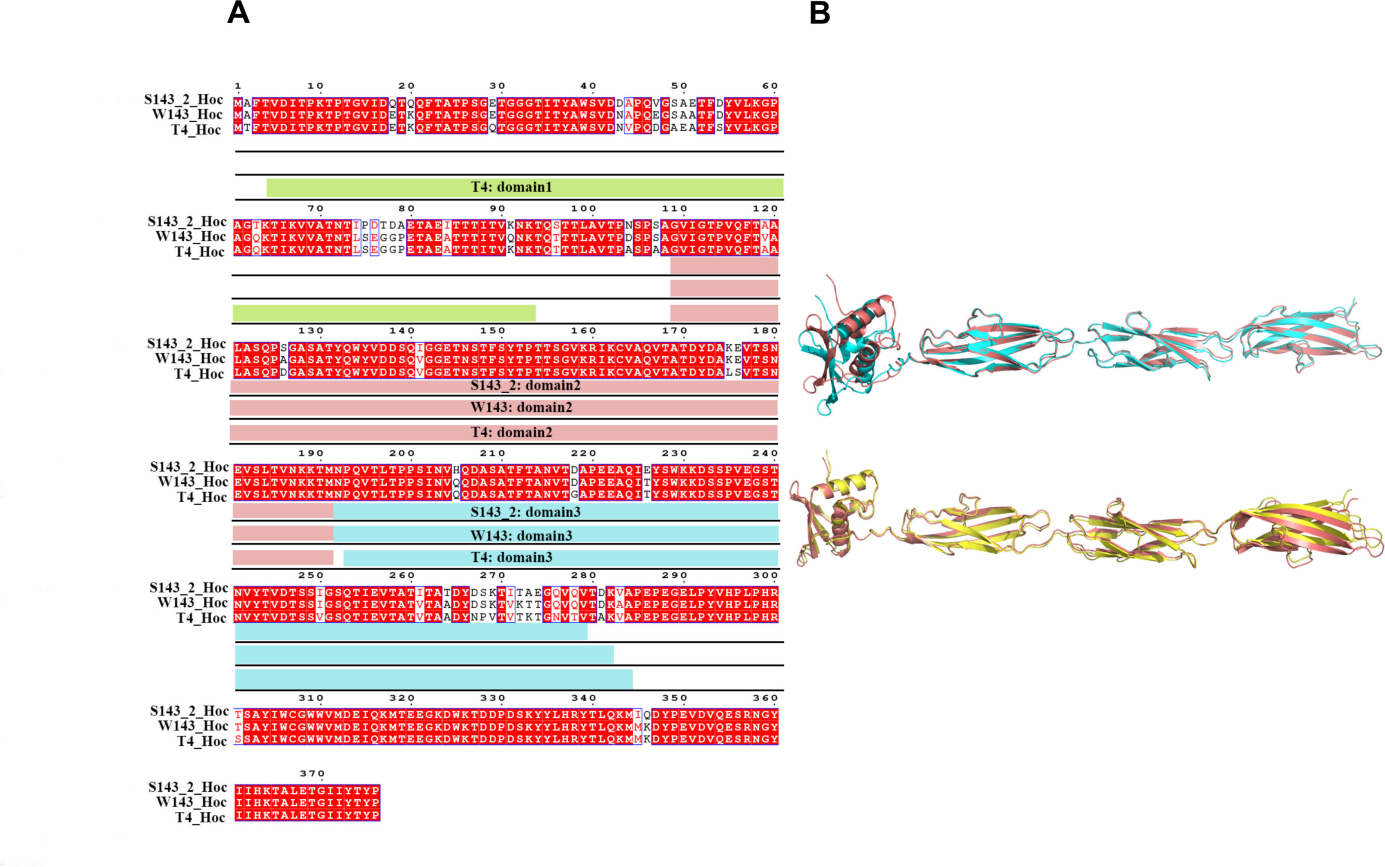
Sequence and structural comparison of Hoc proteins from phages S143, W143, and T4. (**A**) Sequence alignment of Hoc proteins. (**B**) Structural alignment of Hoc proteins predicted by AlphaFold. Pink represents phage T4; green represents phage S143_2; and yellow represents phage W143.

The ability of two strains of T4-like phages W143 and S143_2 to adhere to intestinal epithelium was investigated. Sequence alignment of the Hoc protein revealed that both S143_2 and W143 aligned over the full length (100% coverage) with the T4 Hoc protein, exhibiting sequence identities of 88.3% and 93.09%, respectively, while sharing 92% identity with each other. Compared to T4, although the amino acid lengths of S143_2 and W143 are consistent with that of T4, T4 possesses an additional non-immunoglobulin-like domain 1. Comparative sequence analysis revealed 5 amino acid variations in domain 2 and 17 amino acid variations in domain 3 among the three phage strains (Fig. 1A). To further evaluate structural conservation, the Hoc proteins of S143_2 and W143 were structurally aligned with that of the T4 phage. The resulting RMSD values were 0.941 for S143_2 and 1.130 for W143, respectively, indicating a high degree of structural similarity among these Hoc proteins (Fig. 1B).

**Fig 2:**
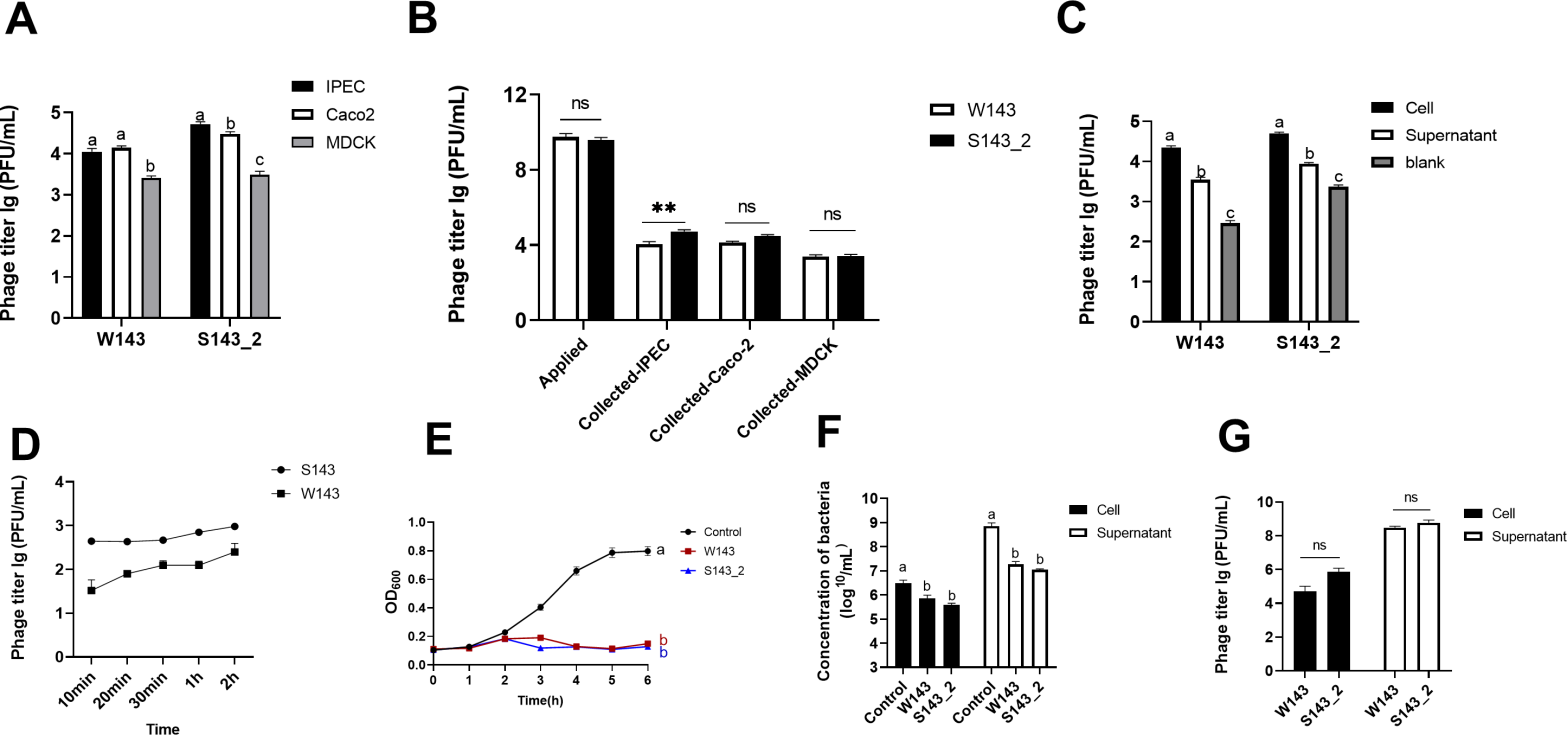
Adhesion capacity and protective effect of different T4-like phages *in vitro*. The applied titers of phages were consistently at 10^10^ PFU/mL, 100 μL. (**A**) Phage titers of W143 and S143_2 adhered to IPEC-1, Caco-2, and MDCK cells. (**B**) Phage titers of W143 and S143_2 in IPEC cells, Caco-2 cells, and MDCK cells. (**C**) Phages were separately inoculated into cells and cell-free blank wells, and 90 min later, non-adherent phages were removed by gently washing the wells five times with PBS. The number of phages in the supernatant from the fifth wash of both blank and cell wells was quantified, along with the number of phages adhering to the cells. (**D**) Phages released from epithelial cells into the supernatant at indicated time points (10, 20, and 30 min, 1 and 2 h) under continuous shaking at 500 rpm. (**E**) Growth curves (OD over time) in the presence (control) or absence of phage (W143 and S143_2). (**F**) Bacterial concentration of IPEC-1 cells and supernatant in control, W143, and S143_2 groups. (**G**) Phage titer of IPEC-1 cells and supernatant in W143 and S143_2 groups. One-way ANOVA followed by Fisher’s LSD multiple comparison test was used to evaluate differences among multiple groups. The *t*-test was applied for comparisons between two groups. Data are shown as mean ± SEM; *n* = 4; ns means *P* > 0.05; **, *P* < 0.01. Groups with the same letter mean *P* > 0.05. Bars with different letters mean *P* < 0.05.

Adhesion assays employing three cell models, including mucus-producing porcine small intestinal epithelial cell line (IPEC)-1 and human colonic epithelial cell line Caco-2 versus non-mucus-secreting Madin–Darby canine kidney (MDCK) cells, were performed by incubating phages with the cells for 1 h, after which non-adherent phages were removed by washing the cells five times, and then attached phages on the cells were determined. The results demonstrated that phages exhibited a significantly higher adhesion efficiency to mucus-secreting cell lines compared to non-mucus-producing cells (Fig. 2A). In IPEC-1 cells, S143_2 exhibited significantly higher adhesion than W143, whereas the adhesion of the two phages to Caco-2 and MDCK cells was comparable (Fig. 2B). In addition, a blank group (cell-free plate) and a cell group (plate with IPEC-1 cells) were prepared, and then the number of phages in the fifth wash supernatant of both groups and the phages adhering to the cells in the cell group were counted. The results showed that (Fig. 2C) the number of both phages adhering to the cells was significantly higher than that in the fifth wash supernatant of the cell group, indicating specific adhesion of phages to the cells rather than residual phages from incomplete washing. Additionally, the phage number in the fifth wash supernatant of the cell group was significantly higher than that of the blank group. Since the blank group represents the residual phages from washing without cells, the higher phage count in the cell group’s supernatant suggests that some phages loosely adhered to the cells were detached during the washing process. These results indicate that the adhesion of phages to epithelial cells is not particularly strong and represents a reversible binding interaction. To further characterize the detachment kinetics of the two phages following adhesion, a dissociation assay was performed. After phage attachment, cells were washed five times with phosphate-buffered saline (PBS) to remove unbound particles, and 1 mL of PBS was added to each well, followed by gentle shaking at 500 rpm. The concentration of phages released into the supernatant was quantified at 10, 20, and 30 min, as well as 1 and 2 h. The results showed that the number of S143_2 phages released from epithelial cells was approximately one order of magnitude higher than that of W143. This observation suggests that increased initial binding results in a greater number of dissociated phages, which may reflect the relatively loose and reversible nature of phage attachment to intestinal epithelial cells. Notably, S143_2 exhibited a slower dissociation rate compared with W143, indicating stronger and more stable adhesion capacity of S143_2 (Fig. 2D). The protective effect against EPEC143 of the two phages after adhesion to the cells was also measured *in vitro*. First, we demonstrated that both phages exhibited comparable lytic activity at a multiplicity of infection of 0.001 in LB liquid medium (Fig. 2E). Then, cells were preincubated with the two phage strains, respectively, for 90 min, and non-adherent phages were removed by washing the cells five times with PBS before exposure to EPEC143 for 4 h. The results showed that both phage groups significantly reduced bacterial counts in cells and supernatants (Fig. 2F and G), with an increase in phage concentration. Although there was no significant difference between the two phages, phage S143_2 exhibited a better bacteriostasis trend.

### Adhesion capacity and protective effect of W143 and S143_2 *in vivo*

**Fig 3:**
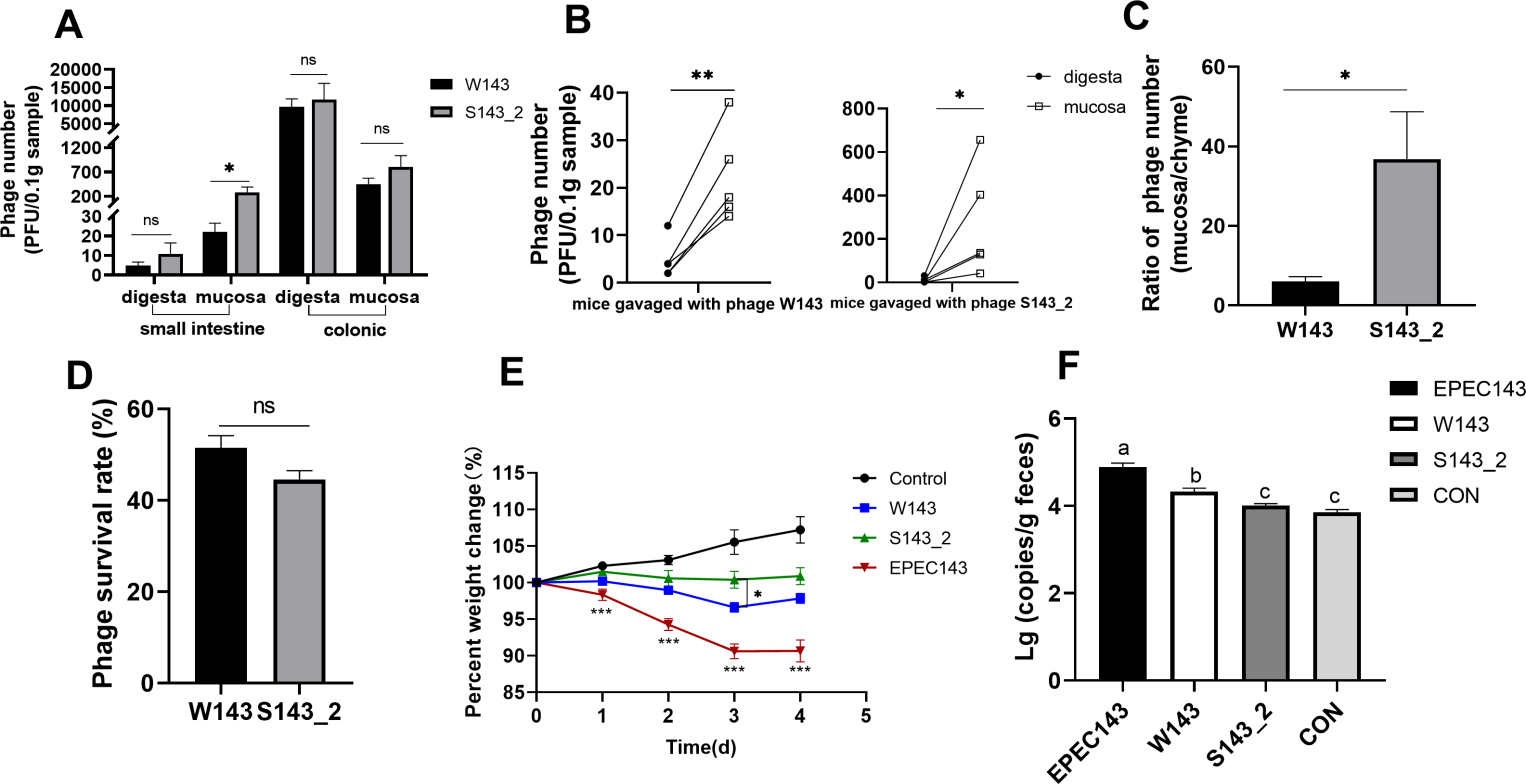
Adhesion capacity and protective effect of different T4-like phages *in vivo*. (**A**) The numbers of phages W143 and S143_2 in small intestinal digesta and mucosa, as well as colonic digesta and mucosa, at 8 h, per 0.1 g sample. (**B**) The numbers of W143 or S143_2 in small intestinal digesta and mucosa at 8 h per 0.1 g sample. (**C**) Ratio of phage number adhering to mucosa and digesta in mice following separate oral administration of W143 and S143_2. (**D**) Survival rate of two phage strains at pH 3 for 90 min. (**E**) Percent weight change of mice following oral administration of SM buffer (control), W143, S143_2 (10^8^ PFU), or EPEC143. Body weight on day 0 was normalized to 100%, and subsequent weight changes were calculated relative to this baseline. (**F**) The number of EPEC143 copies in mouse feces was quantified using quantitative real-time PCR targeting the *escV* gene of EPEC. One-way ANOVA followed by Fisher’s LSD multiple comparison test was used to evaluate differences among multiple groups. *t*-Test was applied for comparisons between two groups. Data are shown as mean ± SEM; *n* = 5; *, *P* < 0.05; **, *P* < 0.01; ***, *P* < 0.001. Groups with the same letter mean *P* > 0.05. Bars with different letters mean *P* < 0.05.

To assess the intestinal mucosal adhesion capability of phages S143_2 and W143 *in vivo*, mice were orally administered with either phage strain. Eight hours post-gavage, animals were euthanized, and small intestinal mucosa samples were collected for phage quantification. This time point was selected based on preliminary data on other phages (data not shown), demonstrating that ingested phages had predominantly migrated to the colon by this time, thereby minimizing residual phage presence in small intestinal digesta and ensuring more accurate mucosal adhesion measurements. Here, our results (Fig. 3A) also showed that at 8 h post-gavage, phage levels in colonic digesta were markedly higher than those in the small intestine, with phage counts in small intestinal digesta being extremely low. Phage counts in small intestinal mucosa were significantly higher than those in digesta for both treatment groups (Fig. 3B), confirming the mucosal adhesion capability of both phages. Notably, S143_2 exhibited a higher mucosal phage load than W143. For a more precise comparison, the mucosa-to-digesta phage ratio was used as a standardized adhesion index, which was significantly higher in the S143_2 group (Fig. 3C). To exclude the possibility that this difference was due to superior acid resistance of S143_2, we compared the survival of both phages at pH 3 for 90 min as well as their loads in the large intestine and found no significant differences between groups (Fig. 3A and D). These findings collectively demonstrate that the higher mucosal phage count of S143_2 is attributable to enhanced adhesion capacity rather than differential gastric survival.

The protective efficacy of the two phages was further validated. Mice were orally administered 10^8^ PFU of phages at 0 h, followed by oral gavage of 10^8^ CFU of EPEC143 at 4 and 24 h. Mouse body weights were then monitored daily for four consecutive days. It was observed that body weights in all three groups (W143, S143_2, and EPEC143) decreased, especially in the EPEC143 group. Weight loss induced by EPEC143 challenge was effectively alleviated in the phage-pretreated groups, with the alleviating effect in the S143_2 group being significantly larger than that in the W143 group on day 3 (Fig. 3E). Additionally, phage pretreatment was found to effectively reduce the number of EPEC143 strains in feces, with the inhibitory effect of S143_2 being significantly stronger than that of W143 (Fig. 3F). Collectively, these findings demonstrate that phage pretreatment confers protection against EPEC143 infection, with S143_2 providing superior protective efficacy relative to W143.

### Effects of W143 and S143_2 on mouse immunity

**Fig 4:**
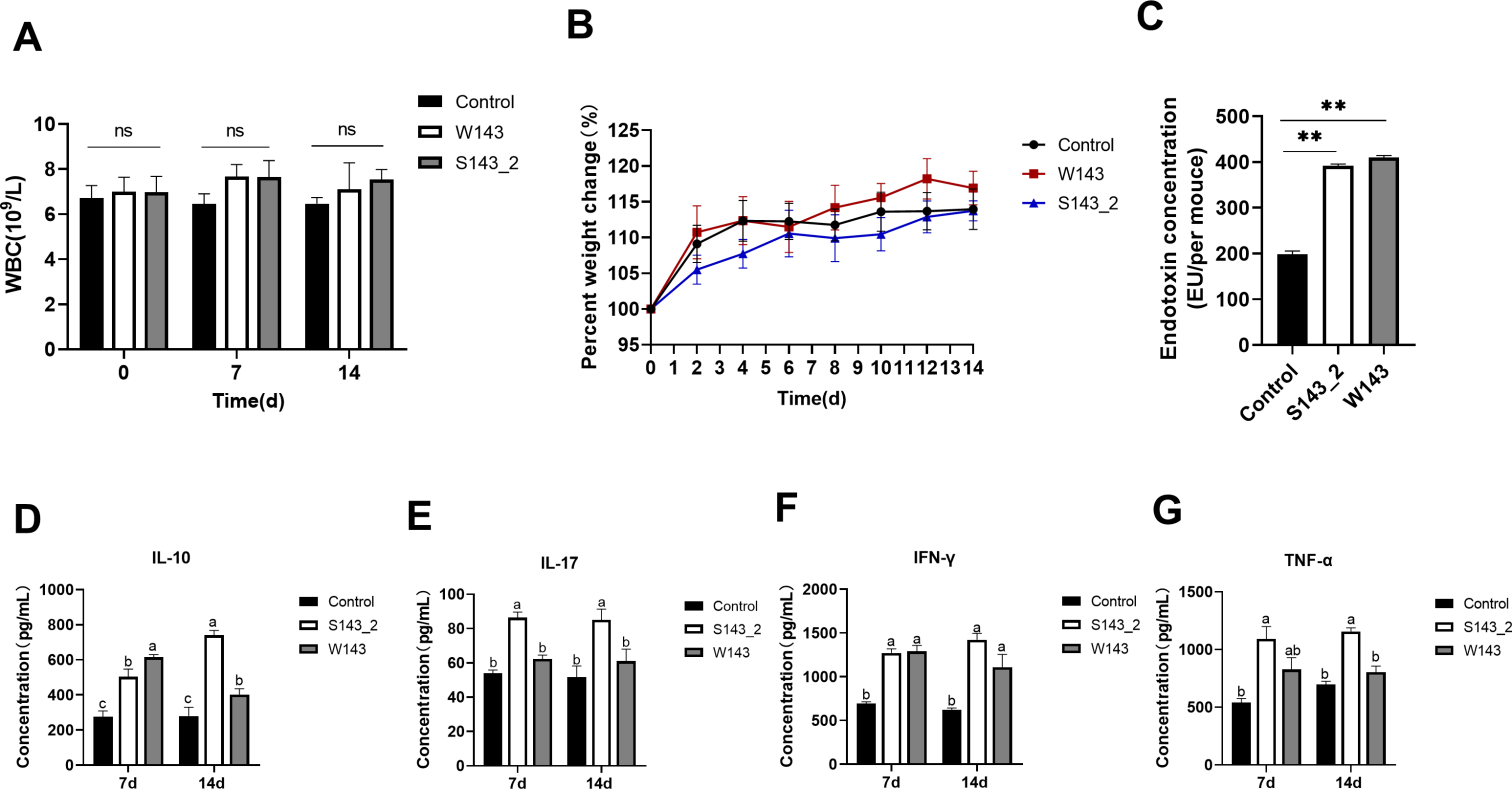
Effects of two T4-like phages on mouse immunity. The total phage dose administered was 10^8^ PFU. (**A**) Number of white blood cells. (**B**) Percent weight change of mice following oral administration of SM buffer (control), W143, and S143_2 (10^8^ PFU). Body weight on day 0 was normalized to 100%, and subsequent weight changes were calculated relative to this baseline. (**C**) Total daily endotoxin intake per mouse in the control and phage-treated (S143_2 and W143) groups. (**D–G**) Serum concentrations of IL-10, IL-17, IFN-γ, and TNF-α in mice. Abbreviations: IFN-γ, interferon-γ; IL-10, interleukin-10; IL-17, interleukin-17; TNF-α, tumor necrosis factor-α. One-way ANOVA followed by Fisher’s LSD multiple comparison test was used to evaluate differences among multiple groups. Data are shown as mean ± SEM, *n* = 6. Groups with the same letter means *P* > 0.05. Bars with different letters mean *P* < 0.05.**, *P* < 0.01.

As shown in Fig. 3A, the phage titers in colonic digesta were significantly lower than those of the administered phages, suggesting limited persistence or replication in the intestinal tract. While oral administration of phages can protect against EPEC143 invasion, the potential impact of phages on mice’s immunity is unclear. Therefore, W143 and S143_2 at a dose of 10^8^ PFU were given via gavage to mice to evaluate their potential immunostimulatory effects. Neither W143 nor S143_2 significantly affected the white blood cell (WBC) count of mice (Fig. 4A). During the initial phase of gavage with phage S143_2, mice exhibited a decreasing trend in body weight compared to the control group; however, as time progressed, their body weight eventually became comparable to that of the control group (Fig. 4B). Endotoxin measurements showed that the endotoxin concentration in the drinking water was 39.7 ± 1.38 EU/mL, whereas the endotoxin levels in 1 mL of phage suspensions (10⁹ PFU) were 1,930 ± 39.9 EU/mL for S143_2 and 2,113 ± 46 EU/mL for W143. Assuming an average daily water intake of approximately 5 mL per mouse, the total endotoxin intake from drinking water alone was estimated to be 198.5 ± 6.90 EU per mouse. In the treatment groups, mice were gavaged with 100 μL of phage suspension (1 × 10⁹ PFU/mL). When combined with endotoxin intake from drinking water, the total daily endotoxin exposure reached 391.5 ± 3.99 EU per mouse in the S143_2 group and 409.8 ± 4.6 EU per mouse in the W143 group. Although the endotoxin intake of the experimental group was significantly higher than that of the control group, given that endotoxin concentrations in the murine small intestinal lumen are typically on the level of 10³−10⁵ EU/mL (16), the additional endotoxin introduced via gavage represents a relatively minor contribution (Fig. 4C). In the S143_2 group, serum concentrations of all inflammatory cytokines were significantly higher compared to the control group; by contrast, in the W143 group, only interleukin-10 (IL-10) and interferon-γ (IFN-γ) levels were significantly elevated relative to the control group. Notably, on day 14, concentrations of IL-10, IFN-γ, interleukin-17 (IL-17), and tumor necrosis factor-α (TNF-α) in the S143_2 group were collectively significantly higher than those in the W143 group (Fig. 4D through G). These findings demonstrate that S143 induces a more robust immune response in the host compared to W143.

**Fig 5:**
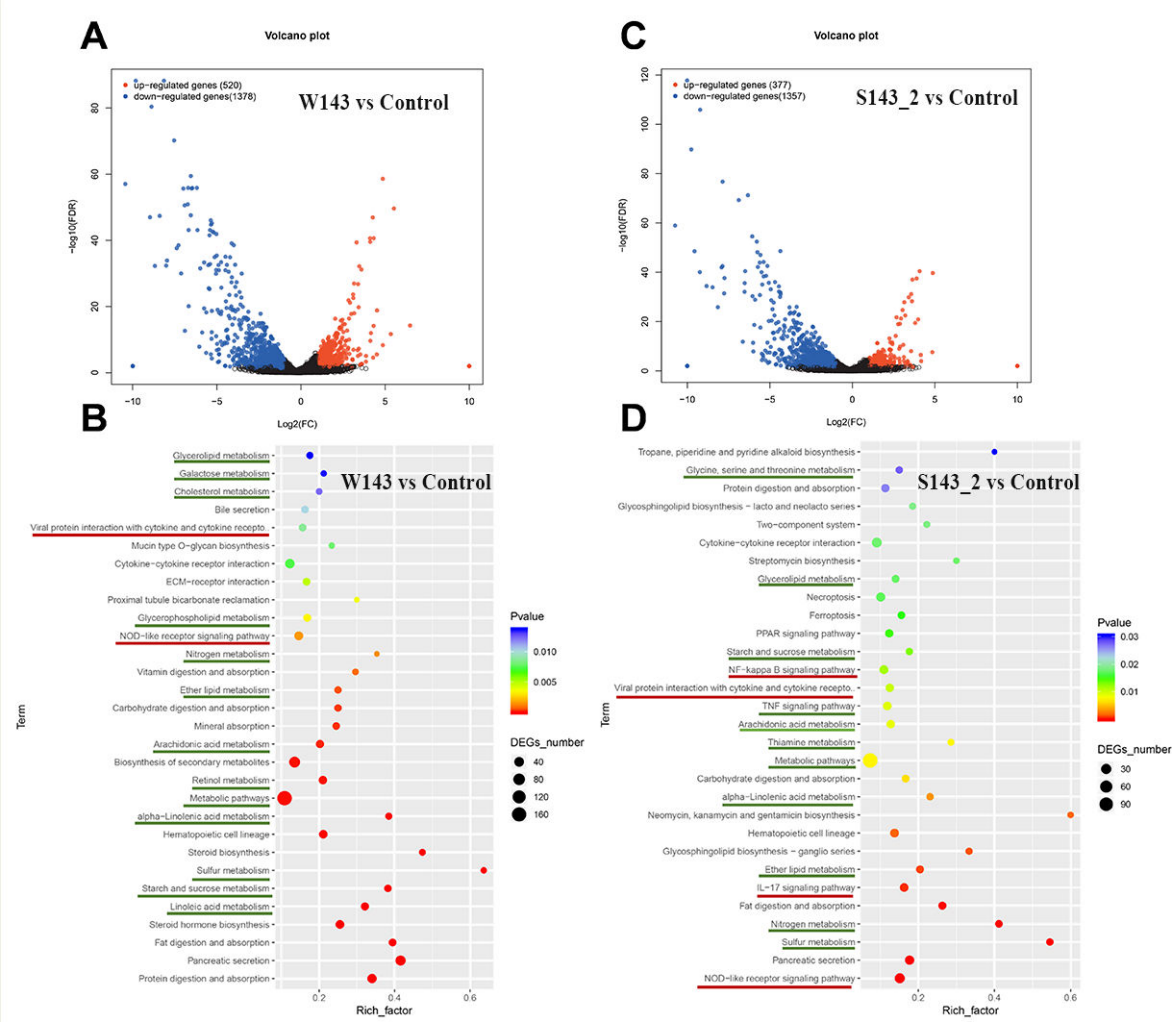
Transcriptome sequencing of small intestinal mucosa after 14 days of gavage with W143 and S143. (**A**) Volcano plot of differentially expressed genes (DEGs) between the control and W143 groups. (**B**) Kyoto Encyclopedia of Genes and Genomes (KEGG) pathways of the W143 group. (**C**) Volcano plot of DEGs between the control and S143_2 groups. (**D**) KEGG pathways of the S143_2 group. Green and red underlines indicate metabolic pathways and immune-related pathways, respectively. Data are shown as mean ± SEM, *n* = 3.

RNA sequencing was also conducted on small intestinal mucosal tissues. As illustrated by the volcano plot, under the criteria of *P* < 0.05 and |log_2_(fold change)| ≥ 2, a total of 520 genes were upregulated and 1,378 genes were downregulated in the W143 group following phage gavage (Fig. 5A), whereas the S143_2 group exhibited 377 upregulated genes and 1,357 downregulated genes (Fig. 5B). Kyoto Encyclopedia of Genes and Genomes (KEGG) pathway analysis revealed that the differentially expressed genes (DEGs) in both the W143 and S143_2 groups were primarily enriched in metabolic pathways; however, the S143_2 group exhibited higher enrichment in immune-related pathways compared with the W143 group (Fig. 5D).

### Two phage strains affect the composition of gut microbiota in mice

**Fig 6:**
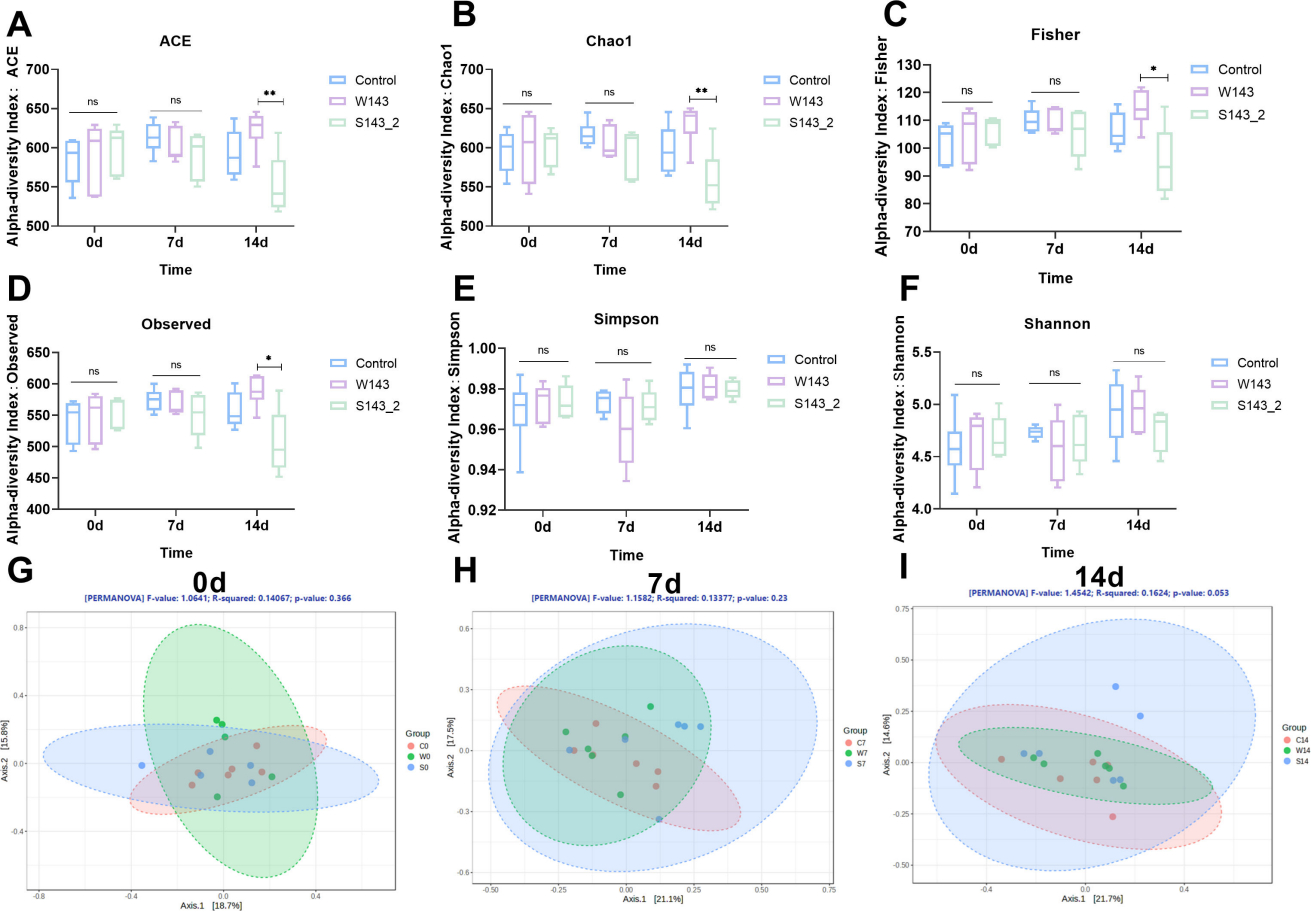
α-Diversity analysis and principal coordinate analysis (PCoA) of mice fecal microbiota. (**A**) ACE index, (**B**) Chao1 index, (**C**) Fisher index, (**D**) observed index, (**E**) Simpson index, and (**F**) Shannon index of mice fecal microbiota between control, W143, and S143_2 groups on days 0, 7, and 14. (**G**) PCoA of samples taken from the control, W143, and S143_2 groups on day 0. (**H**) PCoA of samples taken from the control, W143, and S143_2 groups on day 7. (**I**) PCoA for control, W143, and S143_2 groups on day 14. The Kruskal–Wallis test was used to analyze microbial diversity. Data are shown as mean ± SEM, *n* = 6; ns means *P* > 0.05; *, *P* < 0.05; **, *P* < 0.01.

The principal coordinate analysis revealed no significant differences in microbial community structure among the control, W143, and S143_2 groups on days 0, 7, and 14 (Fig. 6G through I). However, following 14 days of continuous intragastric administration, significant differences were observed in α-diversity between the W143 group and the S143_2 group in terms of the ACE index, Chao1 index, Fisher index, and observed index *(*Fig. 6A through F).

**Fig 7:**
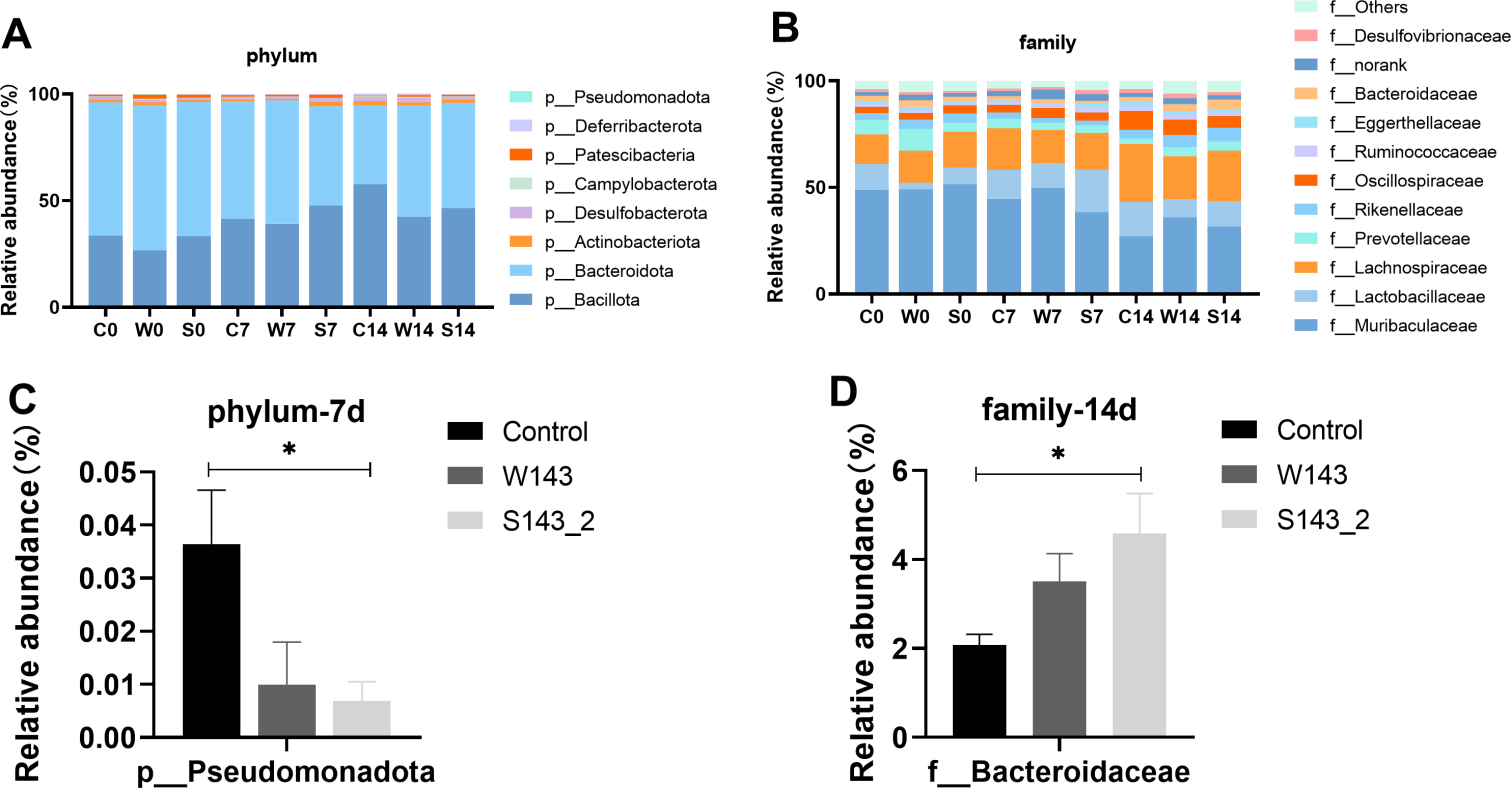
Microbial composition of mice feces among control, W143, and S143_2 groups on days 0, 7, and 14. (**A**) Relative abundance of bacteria at the phylum level in mice feces. (**B**) Relative abundance at the microbial family level. (**C**) Abundance of *Pseudomonadota* on day 7. (**D**) Abundance of *Bacteroidaceae* on day 14. The Kruskal–Wallis test was used to analyze the relative abundance of bacteria. Data are shown as mean ± SEM; *n* = 6; *, *P* < 0.05.

At the phylum level, *Bacillota* and *Bacteroidota* were the dominant taxa (Fig. 7A). At the family level, *Muribaculaceae*, *Lactobacillaceae*, and *Lachnospiraceae* were most predominant (Fig. 7B). On day 7, the relative abundance of *Pseudomonadota* in both groups displayed a downward trend compared to the control group, with the S143 group showing statistical significance (Fig. 7C). On day 14, the relative abundance of *Bacteroidaceae* in both groups exhibited an upward trend, again with significance observed exclusively in the S143 group (Fig. 7D).

## DISCUSSION

*In vitro* and *in vivo* experiments demonstrated differences in adhesive capacity among phage strains, with S143_2 exhibiting stronger adhesion than W143. Extensive studies have indicated that Hoc proteins containing immunoglobulin-like domains are involved in the adhesion mechanism of phages (5, 6, 17, 18). The differences in adhesive capacity between the two phage strains may stem from variations in their Hoc proteins. Fu et al. reported that the *Escherichia coli* phage ΦPNJ-9 lacks domain 3 yet retains the ability to adhere to the mucosa, with residues S183, L184, and T185 in domain 2 being critical for interaction with the MUC2 protein (8). Notably, both W143 and S143_2 also harbor these residues (S183, L184, and T185) within domain 2. However, given the markedly distinct adhesion phenotypes of the two strains despite sharing these confirmed key residues, we propose two non-mutually exclusive explanations for this observation: (i) in addition to these known critical residues, other variable residues that differ between the Hoc proteins of W143 and S143_2 may also contribute to modulating adhesion ability; (ii) other phage-encoded proteins besides Hoc may be involved in regulating the adhesion process, leading to the observed phenotypic differences between the two strains.

We observed that phage S143_2 exhibited stronger adhesion than W143 to IPEC-1 cells, whereas no such difference was detected in Caco-2 cells. This discrepancy is likely attributable to differences in mucus production and glycosylation patterns between the two epithelial cell lines. IPEC cells exhibit mucus-associated features, including the expression of membrane-bound mucins and glycoproteins that contribute to host–microbe interactions (19). In contrast, Caco-2 cells produce limited mucus under monoculture conditions and display a less complex glycosylation profile, which may reduce their ability to mimic the native mucus layer unless co-cultured with goblet-like cells such as HT29-MTX (20). The reduced mucus production and altered glycosylation patterns in Caco-2 cells likely limit the availability of glycan-binding sites required for differential phage adhesion, which may explain why no significant difference in adhesion between S143_2 and W143 was observed in this cell line.

Numerous previous studies have demonstrated that phage adhesion to epithelial cells confers protective effects (2, 21, 22); however, no research has confirmed whether differences in adhesion capacity lead to variations in protective efficacy. Our *in vivo* experiments revealed that, even in the absence of significant differences in host-lysis abilities among phages, differences in adhesion capacity further resulted in distinct protective capabilities. Therefore, phage adhesion capacity could serve as one of the indicators for evaluating the therapeutic potential of phages in future applications.

Additionally, our results indicate that adhesion of phage to mucosa is in the form of loose attachment. This loose adhesion can be described as a weak, reversible mucus binding rather than a specific receptor-mediated adsorption. This may represent a highly efficient mechanism: when phages do not encounter host bacteria, such loose adhesion increases their residence time in the intestine; once they encounter host bacteria, this loose adhesion may dissociate, allowing phages to detach and bind to the host bacteria receptor, where the binding is irreversible. If adherent phages fail to encounter host bacteria within a certain time frame, their numbers on the mucosa will progressively diminish due to the continuous flow and flushing action of digesta, ultimately resulting in complete elimination.

Notably, when applying phage therapy, a comprehensive evaluation of the phages’ intrinsic impact on the host is also essential. Our study further found that phage S143_2 more potently stimulated the host immune system, leading to elevated levels of immune factors. This phenomenon may be attributed to the greater adhesion of S143_2 to the intestinal epithelium, facilitating increased endocytosis or transcytosis by epithelial cells and subsequent enhanced recognition by the host immune system (23–26). Alternatively, it could be related to the specific protein properties of S143_2 that more effectively trigger immune responses (27, 28). Stronger immune stimulation may work synergistically with physical adhesion to enhance protection during bacterial challenge, supported by previous reports. Zamora et al. demonstrated that lytic phages can adhere to bronchial epithelial cells and be sensed by the epithelium, leading to the induction of antiviral and proinflammatory cytokine secretion (29). Similarly, Varadan and Grasis reported that the filamentous phage M13 can induce interleukin-8, TNF-α, interferon beta, and interferon lambdaexpression in HT-29 intestinal epithelial cells, and reduce bacterial internalization compared with controls (30). Additionally, even when administered phages do not proliferate in the gut, alterations in the mouse intestinal microbiota were still observed. This may also be due to phage-induced changes in immune status following gavage, which in turn affect the abundance of specific intestinal microbiota (31–33). Similar results were obtained in our previous phage gavage experiments in pigs (34). Currently, we cannot definitively conclude whether this immune system stress is predominantly beneficial or detrimental. We also hypothesize that the greater weight loss trend observed in the S143_2 group during the early stage of gavage may be associated with immune stress induced by S143_2 stimulation; however, the stress gradually subsided several days post-gavage, and the body weight eventually caught up with that of the control group.

Conclusively, our results revealed that two different T4-like phages with 92% identity in their Hoc proteins exhibited significant differences in adhesive capacity. Such variations in adhesion may further contribute to divergent abilities in protecting intestinal mucosa against pathogenic bacterial invasion. Furthermore, two T4-like phages also show significant differences in regulating the host’s immune response. Therefore, when utilizing phage therapy for bacterial infections, evaluation should not be limited to host-lysis abilities alone; equal consideration must be given to phage adhesive capacity and their ability to regulate host immune function.

## MATERIALS AND METHODS

### Bacteria and phages

Two T4-like phages, W143 and S143_2, capable of infecting enteropathogenic *E. coli* (EPEC), and their common host strain EPEC143, were previously isolated in our laboratory.

### Phage stock preparation

Phage amplification was performed using EPEC143. The amplified phage stock was first filtered through a 0.22 μm filter for sterilization, followed by ultrafiltration twice using a 300 kDa ultrafiltration tube to reduce endotoxin levels. Endotoxin levels in the purified phage preparation were determined using an endotoxin assay kit (GenScript, USA), with three replicates set for each sample. All operations were performed in strict accordance with the manufacturer’s instructions. The phage titer was determined using the double-layer agar plaque assay.

### Phage adhesion and host-lysis activity *in vitro*

For phage adhesion assays, IPEC-1 cells, human colonic epithelial cell line Caco-2, and MDCK cells were seeded into 12-well plates at a density of approximately 1 × 10^5^ cells per well. When cells reached 90%–100% confluence, each well was incubated with W143 or S143_2 at 10^10^ PFU/mL (100 μL) for 90 min at 37°C, 5% CO_2_. After incubation, non-adherent phages were removed by washing the cells five times with 1 mL PBS per cell. Subsequently, cells were scraped into 1 mL of PBS, and the adherent phages were then quantified using the double-layer agar plaque assay. To investigate the adhesion mechanisms of phages, the same cell seeding and phage incubation procedures were performed as described above. The supernatants from five consecutive PBS washes of IPEC-1 cells were collected and quantified for phage titers. Additionally, the number of phages adhering to the cells was determined. As a control, the same procedure was performed using cell-free culture dishes, and the phage titers in the supernatants after five gentle PBS washes were also measured.

For host-lysis activity tests, phages W143 and S143_2 were added to IPEC-1 cells at 10^9^ PFU/mL (100 μL) and incubated for 90 min. Non-adherent phages were removed by washing the cells five times with PBS. Subsequently, a suspension of EPEC143 cells was added to the cells at 10^8^ CFU/mL and incubated in the incubator for 4 h. The concentrations of EPEC143 cells, W143, and S143_2 in the supernatant and those adhering to the IPEC cells were then quantified.

### Comparison of phage detachment rates

IPEC cells were seeded in 12-well plates and cultured to 90%–100% confluence. Phages (10^9^ PFU) were added and co-incubated with the cells for 90 min. After incubation, the cells were washed five times with PBS to remove unbound phages. Subsequently, 1 mL of PBS was added to each well, and the plates were placed on a shaker at 500 rpm to allow gradual detachment of cell-associated phages. At 10, 20, and 30 min and 1 and 2 h, 200 μL of the supernatant was collected from each well. The titer of phages released from the cells was quantified using the double-layer agar plate method to evaluate the detachment rates.

### Animal experiment

Six-week-old male BALB/c mice were purchased from the Comparative Medicine Center of Yangzhou University, Yangzhou, China.

### Phage adhesion to small intestinal mucosa *in vivo*

Ten mice were divided into two groups: the W143 group and the S143_2 group, with five mice in each group. Each mouse was orally administered 10^8^ PFU of the respective phage. After 8 h, the small intestinal mucosa and digesta were collected. After digesta collection, the small intestine was incised and rinsed three times with normal saline to remove non-adherent phages. For the determination of mucosa-associated phages, the intestinal mucosa was carefully scraped using a sterile glass slide and transferred into preweighed sterile tubes containing 1 mL of SM buffer. The samples were then vortexed vigorously for 1 min to detach phages from the mucus layer. The resulting suspension was centrifuged at 8,000 × *g* for 10 min at 4°C to remove cellular debris, and the supernatant was filtered through a 0.22 μm filter.

Phage titers in both digesta and mucosal samples were determined using the double-layer agar plaque assay. Briefly, serial 10-fold dilutions of each sample were prepared in SM buffer, mixed with the host bacteria in semisolid culture, and overlaid onto LB agar plates. After incubation at 37°C for 8 h, plaques were counted, and the results were expressed as PFU per gram of sample.

### Phage antimicrobial activity assay *in vivo*

Twenty mice were randomly assigned to four groups (*n* = 5 per group): control, EPEC143, W143, and S143_2. At 0 h, mice in the phage-treated groups (W143 and S143_2) received an oral gavage of 10^8^ PFU of phages, while those in the control and EPEC143 groups were administered an equal volume of SM buffer used for phage storage. At 4 and 24 h (day 1) after phage administration, both the phage-treated and EPEC143 groups were orally challenged with 10^8^ CFU of EPEC143, whereas the control group received an equal volume of PBS. Body weight was monitored daily. On day 4 post-phage administration, fecal samples were collected for quantitative analysis of EPEC143. Quantification of EPEC143 in fecal samples was conducted using quantitative real-time polymerase chain reaction targeting the secretion system component V (*escV*) virulent gene of EPEC. A plasmid-based standard curve was constructed to facilitate accurate quantification.

### Effect of phages on the immune system

Eighteen mice were divided into three groups: the control group, the W143 group, and the S143_2 group, with six mice in each group. Mice in the phage groups received daily oral administration of phage W143 or S143_2 (10^8^ PFU) from days 1 to 14, while the control group was administered an equivalent volume of SM buffer. The body weights of the mice were measured every 2 days to monitor changes. On days 0, 7, and 14, blood samples were collected via the submandibular vein. After serum separation, the samples were centrifuged at 800 × *g* for 15 min, and the supernatant was stored at −20℃ for further analysis. On day 15, all mice were euthanized, and samples of the small intestinal mucosa and colonic digesta were collected and stored in liquid nitrogen for subsequent experiments.

### Enzyme-linked immunosorbent assay

All reagents and samples were brought to room temperature (21–25°C) before analysis. Mouse enzyme-linked immunosorbent assay kits were used to assay serum concentrations of IL-17, TNF-α, IFN-γ, and IL-10 on days 7 and 14 according to the instructions provided by the manufacturers.

### WBC count

Blood samples collected from mice were sent to Jiangsu Provincial Hospital of Traditional Chinese Medicine for determination of total leukocyte counts using an automated hematology analyzer.

### Fecal DNA extraction and 16S rRNA gene sequencing for microbial analysis

Total genomic DNA was extracted using the OMEGA DNA kit according to the manufacturer’s protocol and then sent to Shanghai Lengen Biotechnology Co., Ltd., for sequencing. The V3–V4 hypervariable regions of the 16S rRNA gene were amplified using universal primers 341F (5′‐ACTCCTRCGGGAGGCAGCAG‐3′) and 806R (5′‐GGACTACCVGGGTATCTAAT‐3′). The resulting sequence data were analyzed for microbial composition at the phylum and family levels, as well as α-and β-diversity, using an online bioinformatics platform (https://www.microbiomeanalyst.ca/MicrobiomeAnalyst/ModuleView.xhtml). The relative abundance of different bacterial taxa was expressed as percentages.

### RNA extraction and transcriptome sequencing of small intestinal mucosa

Fifty milligrams of small intestinal tissue from mice on day 14 was weighed and rapidly homogenized to a fine powder using liquid nitrogen in a mortar. Total RNA was extracted from the tissue using the RNA Extraction Kit (OMEGA, USA) according to the manufacturer’s instructions. RNA concentration and purity were measured using a NanoDrop ND-1000 spectrophotometer (Thermo Fisher Scientific), with OD260/280 ratios between 1.9 and 2.1 considered acceptable.

The total RNA was sent to Shanghai Lingen Biotechnology Co., Ltd., for transcriptome sequencing. Sequencing libraries were constructed starting with 1 μg of total RNA. Poly(A) mRNA was enriched using oligo(dT)-coupled magnetic beads, followed by fragmentation via ion interruption. Double-stranded cDNA was synthesized, end-repaired, adenylated at the 3′ ends, and ligated with indexed adapters. After end repair, A-tailing, and adapter ligation, libraries were amplified by PCR for 15 cycles. Quantified libraries were pooled in equimolar ratios for sequencing. Libraries were sequenced on the Illumina NovaSeq 6000 platform (150 bp*2). Trimmomatic with parameters (SLIDING WINDOW: 4: 15 MINLEN: 75) (v0.36, http://www.usadellab.org/cms/index.php?page=trimmomatic) was employed to trim and quality control the raw paired‐end reads, then the clean reads were separately aligned to the reference genome with orientation mode using HISAT2 (https://daehwankimlab.github.io/hisat2/) software. This software was used to map with default parameters. The reference genome and annotation files (Sus scrofa, GCF_000003025.6) were obtained from the NCBI database. The quality assessment of these data was taken by qualimap_v2.2.1 (http://qualimap.bioinfo.cipf.es/). Use htseq (https://htseq.readthedocs.io/en/release_0.11.1/) to count each gene’s reads.

To identify DEGs between the two different samples, the expression level for each gene was calculated using the fragments per kilobase of exon per million mapped reads method. R statistical package edgeR (v3.34.1, Empirical Analysis of Digital Gene Expression in R; http://www.bioconductor.org/packages/release/bioc/html/edgeR.html/) was used for differential expression analysis. Genes with a false discovery rate (FDR) of <0.05 and |log_2_(fold change)| ≥1 were considered significantly differentially expressed. Gene Ontology enrichment analysis was performed using the GOATOOLS toolset (https://github.com/tanghaibao/GOatools), and KEGG pathway enrichment analysis was conducted using KOBAS (35) (v3.0, http://bioinfo.org/kobas), with an FDR of <0.05 considered statistically significant.

### Protein sequence and structural alignment

Sequences were aligned using CLUSTALW (https://www.genome.jp/tools-bin/clustalw) with default parameters. The figures were generated using ESPript 3.0 (https://espript.ibcp.fr/ESPript/cgi-bin/ESPript.cgi). The structure of the Hoc protein was predicted using AlphaFold (https://alphafoldserver.com/) and structural alignment of bacteriophage proteins was performed using PyMOL.

### Determination of phage acid stability

A 500 μL aliquot of phage filtrate with a concentration of 10^9^ PFU/mL was mixed with 3 mL of SM buffer at pH 3. The mixture was then incubated at 37°C in a water bath for 90 min, after which phage concentration was determined using the double-layer agar plate method.

### Statistical analysis

SPSS 23.0 was used to analyze microbial diversity and relative abundance of bacteria via the Kruskal–Wallis test. Normality and homogeneity of variance were assessed using the Shapiro–Wilk test and the Levene test, respectively. For normally distributed data with equal variances, one-way ANOVA followed by Fisher’s LSD multiple comparison test was used to evaluate differences among multiple groups. For comparisons between two groups, the Student *t*-test was applied when variances were equal, whereas the Welch *t*-test was used when variances were unequal. *P* < 0.05 was considered statistically significant. Statistical significance was denoted as *P < 0.05, **P < 0.01, and ***P < 0.001. Groups sharing the same letters indicate no significant difference, whereas different letters indicate statistically significant differences. The number of biological replicates (*n*) is provided in the corresponding figure legends. Data visualization was performed using GraphPad Prism 8.0.

## Data Availability

The sequences of phages W143 and S143_2, as well as the 16S rRNA gene sequence of their host strain EPEC143, were deposited in GenBank (accession numbers MZ189262, MZ189261, and MH671463). The RNA-seq data from small intestinal tissues were deposited in the NCBI under accession number PRJNA1463601. The 16S rRNA sequencing data from fecal samples were deposited under accession number PRJNA730634.
